# Beyond Suitability: Mapping Current and Projected Conservation Gaps for Three Rare Pheasants in Karst Landscapes of Southwest China

**DOI:** 10.3390/ani16172753

**Published:** 2026-09-02

**Authors:** Jianpu Wu, Jinyou Yang, Jingcheng Ran, Yun Wang, Junyi Li, Jingyi Zhu, Jun Zou, Xue Gou, Hong Luo, Shaochuan Cheng

**Affiliations:** 1 Guizhou Academy of Forestry, Guiyang 550000, China; jianpuwu@163.com (J.W.);; 2 Key Laboratory for Conserving Wildlife with Small Populations in Yunnan, College of Forestry, Southwest Forestry University, Kunming 650224, China; 3 Qianxinan Bouyei and Miao Autonomous Prefecture Forestry Bureau, Xingyi 562400, China; 4 Guizhou Forestry Bureau, Guiyang 550000, China; 5 Administration of Wildlife and Forest Plants of Guizhou Province, Guiyang 550001, China; 6 Guizhou Provincial Longli State-Owned Forest Farm, Longli 551200, China

**Keywords:** habitat suitability, habitat quality, protected-area representation, conservation gaps, climate change, karst landscape

## Abstract

Climate-driven range shifts may create new environmentally suitable areas, but whether these areas provide favourable habitat conditions or adequate protection remains poorly understood. We assessed conservation gaps for three rare pheasants (*Syrmaticus humiae*, *S. ellioti* and *S. reevesii*) in Guizhou Province, southwestern China. Using camera-trap records from 1573 sites surveyed from January 2023 to January 2026, we integrated MaxEnt modelling, 2041–2060 climate projections, InVEST-based relative habitat-condition assessment and protected-area overlays. Dominant predictors differed among species: *S. humiae* and *S. reevesii* were mainly associated with climatic factors, whereas *S. ellioti* was more related to soil organic carbon, karst rocky desertification and the Human Footprint Index. Projected potential suitable areas increased for all species under future climate scenarios, but most projected expansion areas under SSP126, SSP245 and SSP585 occurred outside protected areas. Predicted high suitability did not always coincide with favourable habitat condition. *S. reevesii* showed the clearest suitability–quality mismatch, whereas *S. ellioti* showed the highest protected-area representation of high-suitability × high-quality areas. Predictions for *S. reevesii* should be interpreted cautiously because of its limited occurrence records and sensitivity to individual records. These findings show that suitability maps alone may misguide conservation priorities and highlight candidate areas for field verification, restoration, protected-area optimization and climate-adaptation monitoring in karst landscapes.

## Simple Summary

Rare pheasants in southwestern China face simultaneous pressures from climate change, habitat degradation and incomplete protected-area coverage. We used records from 1573 camera-trap sites surveyed throughout Guizhou Province between January 2023 and January 2026 to assess three species: *Syrmaticus humiae*, *S. ellioti* and *S. reevesii*. We modelled their current and projected potential suitability, evaluated habitat condition and degradation risk, and examined whether important areas were represented within existing protected areas. The three species were associated with different environmental factors. Potentially suitable areas increased under the 2041–2060 climate scenarios, but most areas projected to become suitable under SSP126, SSP245 and SSP585 were outside protected areas. Areas predicted to be highly suitable did not always retain favourable habitat condition. *S. reevesii* showed the clearest mismatch between suitability and habitat quality, whereas *S. ellioti* had the greatest protected-area representation of high-suitability areas with high habitat quality. Predictions for *S. reevesii* should be interpreted cautiously because of its limited occurrence records. These findings show that suitability maps alone are insufficient for conservation planning and that habitat condition, degradation risk and protected-area representation should be considered together when identifying candidate areas for protection, restoration and future monitoring.

## 1. Introduction

Conservation planning in the face of global change must move beyond mapping where species occur today to secure their long-term persistence. Identifying environmentally suitable areas is a necessary starting point; however, many of these areas are already degraded, poorly protected, or ill-matched to the shifting requirements of species. Therefore, systematic conservation planning aims to locate refugia—places that not only capture current biodiversity but can also sustain viable populations under ongoing environmental pressure [1,2]. This task is becoming increasingly urgent as climate change, rapid land-use conversion, road expansion, and severe habitat fragmentation converge to reshape wildlife habitats, eroding climate suitability, shrinking core areas, amplifying edge effects, and isolating remnant patches [3,4,5,6,7]. Roads warrant particular attention because their ecological footprint extends well beyond the road surface, elevating human disturbance, increasing mortality risk, and opening remote wilderness to exploitation [8]. These pressures are especially acute in mountain landscapes, where steep environmental gradients, constrained dispersal corridors, and naturally fragmented terrain heighten species’ vulnerability [5,9]. The central challenge for planners is to determine whether climatically suitable areas also have robust habitat quality, face manageable levels of degradation, and are within functioning protected area networks.

Spatial predictions from species distribution models (SDMs) offer a powerful starting point for this assessment, but their conservation value depends on how carefully they are aligned with conditions on the ground. SDMs identify the environmental drivers that shape a species’ current potential range and can project how this range may shift under future climate scenarios [10]. Maximum entropy (MaxEnt) modelling has become a workhorse for presence-only data and is especially valuable for rare or cryptic species, although predictions from small sample sizes require cautious interpretation [11,12,13,14]. Translating model output into conservation action requires converting continuous suitability surfaces into binary maps or using them to guide reserve delineation [15]. Practitioners increasingly recognize that models must account for climate-driven range shifts, concurrent land-use changes, and inherent uncertainties; otherwise, conservation investments risk being misdirected [16,17,18]. Suitability maps become genuinely useful for conservation only when they are grounded in real landscape conditions, local threat profiles, and actual reserve coverage rather than relying on idealized bioclimatic envelopes alone.

Bioclimatic suitability does not guarantee a high-quality habitat on the ground, and this disconnect can seriously undermine spatial prioritization. Large expanses identified as highly suitable by macroclimatic models may already be heavily degraded, fragmented or under intense human pressure. Land-use change and associated human impacts remain the dominant drivers of biodiversity loss worldwide, often eroding habitat quality and carrying capacity, even when the broad climate remains favourable [19,20]. To address this mismatch, the InVEST Habitat Quality model provides a spatially explicit, threat-based framework that quantifies landscape degradation and habitat integrity by integrating land cover data with threat layers and land-cover sensitivity values to specified threats [21]. Pairing SDM outputs with this habitat quality metric allows planners to separate areas that are merely macroclimatically suitable from those that retain structural integrity and face low-threat levels. This distinction carries direct practical consequences: highly suitable but degraded areas become priorities for ecological restoration or ground-truthing, whereas intact, highly suitable sites outside existing reserves represent urgent opportunities for protected area expansion or the designation of Other Effective Area-based Conservation Measures (OECMs).

However, the effectiveness of any protected area network depends on how well it represents diverse ecosystems and shields species from future habitat loss. A large body of evidence shows that although the global protected area estate continues to grow, many networks still fall short of protecting endangered species [22,23,24]. Protected areas can reduce habitat loss; however, their performance varies widely with location, management quality, and spatial design [25,26,27]. Moreover, assessments that focus solely on current representation can be deceptive because protection on paper does not ensure long-term viability if the sites themselves are already degraded, climatically unstable, or disconnected from future climate refugia [28]. As climate and land use shift in tandem, critical habitats may slide out from under existing reserve boundaries, opening substantial gaps and decoupling protected area networks from the future needs of species [29,30,31]. Therefore, effective planning demands an integrated lens that simultaneously weighs climate suitability, habitat quality, local degradation risk, and protected area configuration.

Karst landscapes add particular complexity to conservation planning because shallow and discontinuous soils, exposed bedrock, steep terrain and rocky desertification can create highly heterogeneous and fragmented habitat mosaics [32]. In such settings, areas predicted as climatically or environmentally suitable may still differ greatly in habitat condition, degradation risk and protection status, making an integrated conservation assessment especially necessary. In this study, we developed an integrated spatial conservation framework to evaluate the current and future climate suitability, habitat quality, degradation risk, and protected area coverage for three rare pheasants in Guizhou Province, China. Our specific objectives were to (1) model current habitat suitability and identify key environmental drivers for *Syrmaticus humiae*, *Syrmaticus ellioti*, and *Syrmaticus reevesii* using the MaxEnt algorithm; (2) project changes in suitable habitat under 2041–2060 climate scenarios, quantifying areas of expansion, contraction, and stability; (3) assess landscape-level habitat quality and map local degradation risk using the InVEST model; and (4) overlay these outputs with the existing reserve network to pinpoint high-quality suitable habitat, degraded areas, and critical protection gaps. By explicitly linking broad-scale climate suitability with fine-scale landscape conditions and management boundaries, this study provides a reproducible, spatially explicit workflow to guide targeted field verification, habitat restoration, and reserve expansion in karst ecosystems.

## 2. Materials and Methods

### 2.1. Study Area and Target Species

This study was conducted in Guizhou Province, southwestern China (24°37′–29°13′ N, 103°36′–109°35′ E) (Figure S1). The province is dominated by mountains, hills, and dissected plateaus, with elevations generally decreasing from west to east. It has a humid subtropical monsoon climate, with a mean annual temperature of approximately 14–16 °C and annual precipitation of approximately 1100–1300 mm. Guizhou is one of the most typical karst regions in China, where exposed karst covers approximately 61.9% of the provincial area. Steep terrain, carbonate bedrock, thin soils, and uneven water availability create a highly heterogeneous landscape and contribute to ecological vulnerability [33].

The land cover in Guizhou is characterized by a mosaic of forests, shrublands, croplands, grasslands, and built-up areas, with forest and shrub habitats often embedded within agricultural land, settlements, and road networks. This landscape structure provides suitable conditions for evaluating the combined effects of climatic suitability, habitat quality, habitat degradation, and protected area coverage on mountain pheasants.

Three rare pheasant species were selected: *S. humiae*, *S. ellioti* and *S. reevesii*. These species are associated with montane forests, shrublands, and forest–farmland mosaic habitats, but they differ in their occurrence patterns within Guizhou. Previous studies have shown that habitat structure, elevation, temperature, land-use change, and human infrastructure can influence the distribution or habitat conditions of these pheasants [34,35,36,37].

### 2.2. Occurrence Records and Environmental Predictors

Occurrence records were derived from field-based camera-trap surveys conducted across the entire Guizhou Province from January 2023 to January 2026 using UVL7 infrared camera traps (UOVision Technology (Shenzhen) Co., Ltd., Shenzhen, China). A total of 1573 camera-trap sites were included in the province-wide survey network (Figure S1), with each camera-trap site monitored for at least one year. Camera locations were distributed across the study region to sample forested and mountainous environments considered potentially available to the focal pheasants on the basis of previous field surveys and habitat knowledge, rather than being restricted to previously known occurrence localities. All occurrence records used in this study were obtained from these standardized surveys; public occurrence records from biodiversity databases or citizen-science platforms were not incorporated because database searches did not identify additional usable georeferenced records for the three focal species within Guizhou Province. For species distribution modelling, a camera-trap site was considered an occurrence location when at least one independent detection of a target species was recorded, and repeated detections of the same species at the same site were treated as a single record. To reduce spatial clustering, sampling bias and spatial autocorrelation, occurrence records were spatially thinned so that only one record was retained within each 1 × 1 km grid cell, corresponding to the spatial resolution of the environmental predictors. This procedure was intended to reduce, rather than completely eliminate, potential bias associated with the spatial distribution of occurrence records. After filtering, 65 occurrence sites were retained for *Syrmaticus humiae*, 27 for *S. ellioti* and 9 for *S. reevesii*.

Environmental predictors were selected to represent climatic conditions, topography, vegetation productivity, land cover, soil properties, karst environmental characteristics, and anthropogenic disturbance, all of which may influence habitat suitability of forest-associated pheasants (Table S1). The climatic predictors included annual mean temperature (bio1), temperature seasonality (bio4), annual precipitation (bio12), and precipitation of the driest month (bio14). Current climatic variables were obtained from WorldClim v2.1, and future climatic variables (2041–2060) were derived from CMIP6-based WorldClim projections under SSP126, SSP245, and SSP585 scenarios [38,39,40]. Additional predictors included elevation, slope, terrain roughness, cumulative gross primary productivity (gpp_cum), land use/land cover type (lucc), Human Footprint Index (hfp), distance to roads (disroad), soil organic carbon (soil_oc), soil pH (soil_ph), soil clay content (soil_clay), and karst rocky desertification intensity (rocky).

All predictor layers were clipped to the boundary of Guizhou Province, projected to a common coordinate system, and resampled to a 1 km × 1 km grid. To reduce multicollinearity and improve model interpretability, pairwise Pearson correlation coefficients were calculated for the candidate predictors. When two variables were strongly correlated (|r| > 0.7), one variable was retained based on ecological relevance and interpretability of pheasant habitat use [41]. The resulting predictor set was used for habitat suitability modelling and climate projections. Spatial data preprocessing and raster analyses were performed using ArcGIS Desktop 10.8.

### 2.3. Habitat Suitability Modelling and Future Projections

Habitat suitability was modelled using MaxEnt v3.4.4 [11]. Spatially filtered occurrence records were used as presence data, and 10,000 background points were randomly sampled across Guizhou Province to provide broad representation of the environmental conditions available within the provincial modelling extent while avoiding unnecessary computational redundancy. Models were fitted using cloglog output, a regularization multiplier of 1 and automatic feature selection. Model performance was evaluated using cross-validation and test AUC. Ten-fold cross-validation was used for *S. humiae* and *S. ellioti*. Because only nine spatially filtered occurrence records were available for *S. reevesii*, nine-fold leave-one-out cross-validation was used, with each occurrence record serving once as the held-out test sample and the remaining eight records used for model fitting. Continuous suitability outputs were converted to binary suitable–unsuitable maps using the species-specific 10th percentile training presence threshold, a commonly adopted threshold that reduces the influence of potential location errors and environmental uncertainty while retaining most known occurrence records [42]. Current suitability maps were subsequently classified into unsuitable, low-, moderate- and high-suitability categories for area estimation and conservation analyses. Given the small sample size of *S. reevesii*, prediction sensitivity was further evaluated from variation in the predicted high-suitability area and the spatial standard deviation of suitability predictions across the nine leave-one-out models (Figure S2; Table S10).

To evaluate potential climate-driven changes in habitat suitability, models were projected to 2041–2060 under three CMIP6-based climate scenarios (SSP126, SSP245, and SSP585). For future projections, bioclimatic predictors were replaced with scenario-specific climate layers, whereas relatively stable environmental predictors, including topography, soil properties, and karst rocky desertification, were retained unchanged. Dynamic predictors likely to change substantially in the future, including vegetation productivity, land use/land cover, the Human Footprint Index, and road-related variables, were excluded from future-transfer models because reliable scenario-specific projections were not consistently available. Future projections should therefore be interpreted as changes in potential climatic suitability under static non-climatic constraints rather than as realized distribution shifts. The species-specific threshold derived from the current model was consistently applied to both current and future predictions. Pixel-level transitions between current and future binary maps were classified as contraction, expansion, stable unsuitable, or stable suitable. Stable suitable areas were interpreted as areas of persistent potential climatic suitability, and changes in predicted suitable areas were quantified using net area change, relative change, and habitat persistence, defined as the proportion of currently suitable areas remaining suitable under future conditions.

### 2.4. InVEST Habitat Quality and Degradation Assessment

We assessed landscape-level relative habitat quality and degradation across Guizhou using the InVEST v3.18.0 Habitat Quality model [21,43]. The model estimates habitat degradation and habitat quality by combining land-cover classes, threat layers, threat weights, maximum threat distances, decay functions, and land-cover sensitivity values. We interpreted the outputs as relative land-cover-based habitat conditions for forest-associated pheasants, rather than species-specific realized habitat quality, and subsequently overlaid them with species-specific MaxEnt suitability maps. Accordingly, the same InVEST parameterization was applied to all three species to characterize a common landscape-level habitat-condition background, while species-specific differences were represented by their respective MaxEnt suitability predictions. The land-cover raster was reclassified into seven classes present in the study area: cropland, forest, shrubland, grassland, water, bare land, and built-up land. The InVEST model was run at the native 30 m resolution of the land-cover raster, and all threat layers were projected and rasterized to match this spatial resolution and extent.

Four threat factors were included: built-up land, roads, cropland, and bare/degraded land. These factors represent construction pressure, road-related disturbance, agricultural conversion or matrix effects, and exposed or degraded surfaces. They were selected because the focal pheasants mainly use forest, shrubland, and forest-edge habitats, whereas infrastructure, roads, farmland, and degraded open land may reduce habitat conditions through habitat loss, edge effects, increased human access, and disturbance [8,44,45,46]. Threat weights, maximum threat distances, and decay functions were assigned a priori based on the InVEST user guide, previous habitat-quality applications, and the ecology of mountain pheasants in forest–shrub–farmland landscapes. The full threat parameters and their ecological interpretations are provided in Table S3.

Habitat suitability scores and sensitivity values for each land-cover class were assigned according to the expected ecological roles of forest-associated pheasants (Table S2). Forest and shrubland were assigned high habitat suitability values because they represent primary or forest-edge habitats, whereas grassland and cropland were treated as lower-value matrix habitats, and water, bare land, and built-up land were assigned very low or zero values. Forest and shrubland were also assigned higher sensitivity values to surrounding threats to reflect their expected susceptibility to construction, roads, agricultural conversion, and land degradation. The resulting 30 m habitat-quality and degradation rasters were reclassified into low, moderate, and high classes using the natural breaks method, and class areas were calculated from the 30 m outputs. For overlay analyses with MaxEnt suitability maps and protected areas, the classified InVEST outputs were subsequently aggregated to the same 1 km × 1 km grid used in the habitat-suitability analysis. These classes were used for relative conservation prioritization and should not be interpreted as absolute ecological thresholds for conservation. To evaluate the robustness of the results to uncertainty in user-defined InVEST parameters, we additionally conducted a scenario-based sensitivity analysis using two alternative parameterizations. In the Higher-condition scenario, habitat suitability scores were increased by 20%, whereas threat-sensitivity values, threat weights, and maximum threat distances were decreased by 20%; the opposite adjustments were applied in the Lower-condition scenario. Values constrained to the interval 0–1 were capped at the corresponding bounds, while all other model inputs and settings were held constant. To ensure direct comparability among scenarios, the class thresholds derived from the Baseline model were retained when classifying the Higher- and Lower-condition outputs. Robustness was evaluated by comparing protected-area representation of species-specific high-suitability × high-quality and high-suitability × high-degradation areas among the three parameterizations (Table S9).

### 2.5. Protected-Area Overlay and Conservation Gap Analysis

The protected area (PA) spatial dataset was compiled from the National Platform for Common GeoSpatial Information Services and verified using official and published sources. Nature reserve records were checked against the National Nature Reserve Directory released by the Ministry of Ecology and Environment of China, and other protected area categories were cross-checked with published studies on China’s protected area classification system [47,48]. The final dataset included 327 protected areas, including nature reserves, scenic areas, forest parks, geoparks, and wetland parks. These categories were combined to represent the broad spatial extent of the existing protected-area network while recognizing that they differ in management objectives and protection intensity. No spatial overlaps were present among the compiled protected-area polygons, and therefore no additional dissolving procedure was required. Protected areas were converted to a binary raster with the same projection, extent, and 1 km × 1 km cell size as the suitability, habitat quality, and degradation layers. Raster cells were coded as protected when located within a protected-area boundary and unprotected otherwise.

Protected-area overlays were used to quantify the spatial representation of key habitat categories within the existing protected-area network. For each species, the high-suitability class was intersected with the InVEST habitat quality and degradation classes to identify high-suitability × high-quality, high-suitability × low-quality, and high-suitability × high-degradation areas. Each category was then summarized according to protected-area status to identify areas with relatively high or low representation within the current network and to highlight candidate areas for field verification, restoration, or additional conservation assessment [22,25]. Projected expansion areas under SSP126, SSP245, and SSP585 were also overlaid with protected areas to assess the extent to which projected gains in potential suitability under each 2041–2060 climate scenario were represented within the current protected-area network. Protected-area coverage was calculated as the proportion of each habitat or projected expansion category located within protected areas. All area statistics were calculated from 1 km × 1 km raster cells.

## 3. Results

### 3.1. Model Performance, Environmental Drivers and Current Suitability Patterns

**Figure 1:**
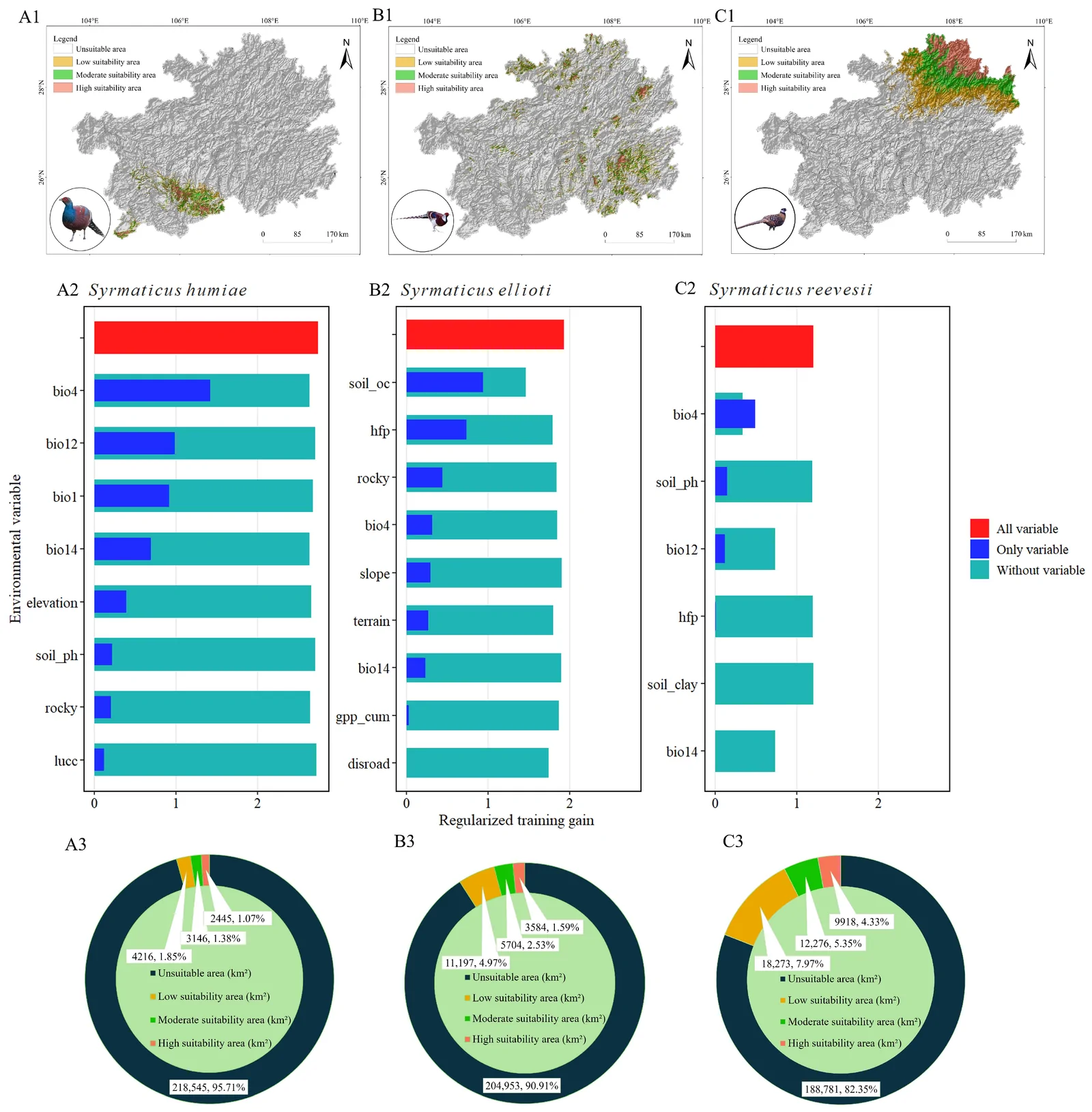
Predicted current habitat suitability, environmental predictor importance and suitability-area composition for three *Syrmaticus* species in Guizhou Province, southwestern China. Columns (**A1**–**C3**) represent *S. humiae*, *S. ellioti* and *S. reevesii*, respectively. Panels (**A1**–**C1**) show predicted current habitat suitability, with grey, yellow, green and red indicating unsuitable, low-suitability, moderate-suitability and high-suitability areas, respectively. Panels (**A2**–**C2**) show MaxEnt jackknife tests based on regularized training gain; red bars indicate the gain of the full model, blue bars indicate the gain when each predictor was used alone, and cyan bars indicate the gain when each predictor was omitted from the model. Panels (**A3**–**C3**) show the area composition of the four suitability classes, with labels indicating area (km^2^) and percentage of the total modelling area.

MaxEnt models showed high discriminatory ability for *Syrmaticus humiae* and *S. ellioti*, with mean test AUC values of 0.969 ± 0.041 and 0.923 ± 0.067. The model for *S. reevesii* also produced a high mean test AUC value (0.903 ± 0.138), although its small sample size warrants cautious interpretation. Predictor importance revealed clear species-specific associations with climatic, edaphic, karst-related, and anthropogenic gradients (Figure 1(A2–C2); Table 1). For *S. humiae*, temperature seasonality was the dominant predictor (bio4, 47.2%), followed by annual mean temperature (bio1, 16.1%), karst rocky desertification (rocky, 11.0%), and elevation (10.8%). Jackknife results showed that bio4 had the highest gain when used alone, whereas omitting the precipitation of the driest month (bio14) caused the largest reduction in training gain. For *S. ellioti*, soil organic carbon was the strongest predictor (soil_oc, 43.4%), followed by karst rocky desertification (18.5%) and the Human Footprint Index (hfp, 13.3%), with soil_oc also producing the greatest reduction in gain when omitted. For *S. reevesii*, climatic variables dominated the model, particularly temperature seasonality (bio4, 51.1%), precipitation in the driest month (bio14, 20.1%), and annual precipitation (bio12, 19.6%).

Predicted current suitable areas differed markedly among the three species in both extent and spatial configuration (Figure 1(A1–C1, A3–C3)). *S. humiae* had the most restricted predicted suitable area, covering 9807 km^2^, or 4.29% of the modelling area. Its predicted high-suitability areas covered 2445 km^2^ (1.07%) and were concentrated mainly around Zhenning, Ziyun, Luodian and Xingyi in southwestern Guizhou. Predicted suitable areas for *S. ellioti* were broader but more fragmented, covering 20,485 km^2^ (9.09%). High-suitability areas for this species covered 3584 km^2^ (1.59%) and were mainly distributed in eastern and northeastern Guizhou, particularly northern Dushan, Leishan, and the border region between Yinjiang and Jiangkou, with additional patches in northern Guizhou, including Chishui, Xishui, Tongzi, Suiyang and Daozhen. *S. reevesii* showed the largest predicted suitable area, covering 40,467 km^2^ (17.65%), with a broad belt of predicted suitability across northern Guizhou. Its high-suitability areas covered 9918 km^2^ (4.33%) and were concentrated mainly around Daozhen, Wuchuan and Yanhe. The leave-one-out analysis indicated moderate sensitivity of the predicted extent for *S. reevesii* to individual occurrence records, with predicted high-suitability areas ranging from 6571 to 14,685 km^2^ (mean = 11,431 ± 3061 km^2^; CV = 26.8%; Table S10), and spatial uncertainty varied across the predicted range (Figure S2). Given the limited number of filtered occurrence records and this model sensitivity, predicted suitable areas for *S. reevesii* should be treated as candidate areas for field verification rather than confirmed core habitats.

### 3.2. Projected Changes in Potential Suitable Areas Under Climate Scenarios

**Figure 2:**
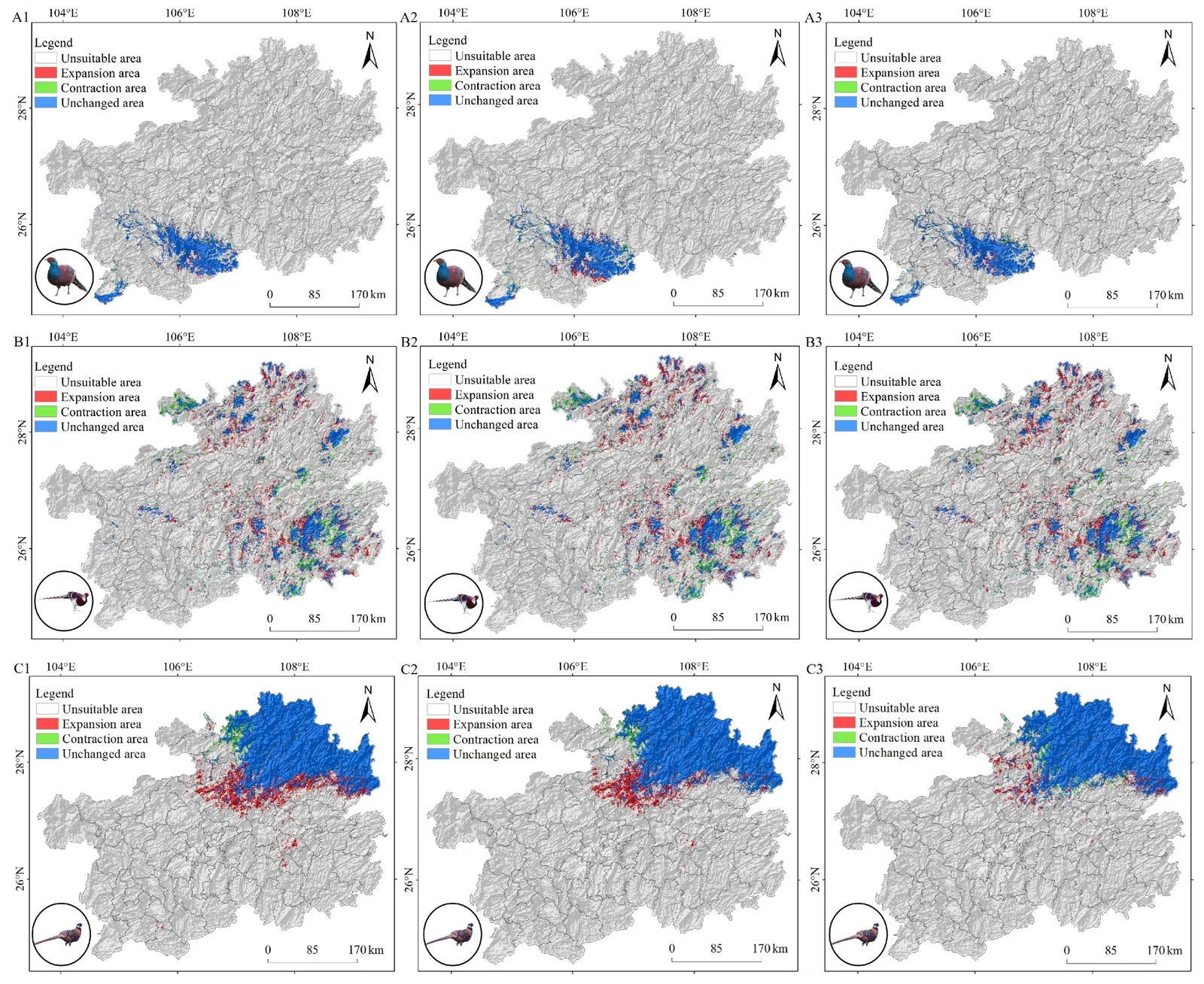
Projected transitions in potential suitable areas for three *Syrmaticus* species in Guizhou Province under mid-century climate scenarios. Transitions between current and 2041–2060 suitability projections are shown under SSP126, SSP245 and SSP585. Rows represent species: (**A1**–**A3**), *S. humiae*; (**B1**–**B3**), *S. ellioti*; and (**C1**–**C3**), *S. reevesii*. Columns represent climate scenarios from left to right: SSP126, SSP245 and SSP585. Grey indicates areas unsuitable in both current and future projections, red indicates projected expansion areas, green indicates projected contraction areas, and blue indicates stable suitable areas relative to the current suitability projection.

Across the 2041–2060 climate scenarios, all three *Syrmaticus* species showed net increases in projected potential suitable area, but the magnitude of change and spatial turnover differed markedly among species (Figure 2; Table S4). These changes represent projected shifts in potential suitability under the climate scenarios rather than confirmed future habitat expansion or occupancy. Overall, *S. humiae* showed the smallest net increase and the most spatially stable pattern, *S. ellioti* showed stronger spatial turnover with both large expansion and contraction areas, and *S. reevesii* showed substantial but more scenario-dependent increases in projected potential suitable area.

For *S. humiae*, stable suitable areas remained the dominant component across all scenarios, ranging from 9215 to 9336 km^2^, and were mainly retained in southwestern and south-central Guizhou (Figure 2(A1–A3)). Projected expansion was limited and occurred mostly along the margins of the current suitable range, with the largest expansion under SSP245 (1528 km^2^). Contraction areas were smaller than expansion areas in all scenarios (429–550 km^2^), resulting in a modest net increase of 510–978 km^2^. In contrast, projected changes for *S. ellioti* were larger and more spatially dynamic. Although stable suitable areas were similar among scenarios (15,411–15,478 km^2^), expansion exceeded 10,000 km^2^ under all scenarios, and contraction was also extensive (5007–5074 km^2^), producing a net increase of 5032–5091 km^2^ (Figure 2(B1–B3)).

*S. reevesii* showed substantial projected increases in potential suitable area, although the magnitude varied considerably among scenarios. Stable suitable areas remained extensive across northern Guizhou (38,760–38,931 km^2^), while projected expansion ranged from 3084 to 7828 km^2^ and was mainly distributed along the margins of the northern suitable belt and in scattered southern patches (Figure 2(C1–C3)). Contraction remained comparatively limited (1018–1189 km^2^), resulting in net increases of 6639 km^2^ under SSP126, 4313 km^2^ under SSP245, and 1940 km^2^ under SSP585.

### 3.3. Relative Habitat Quality and Degradation Patterns

**Figure 3:**
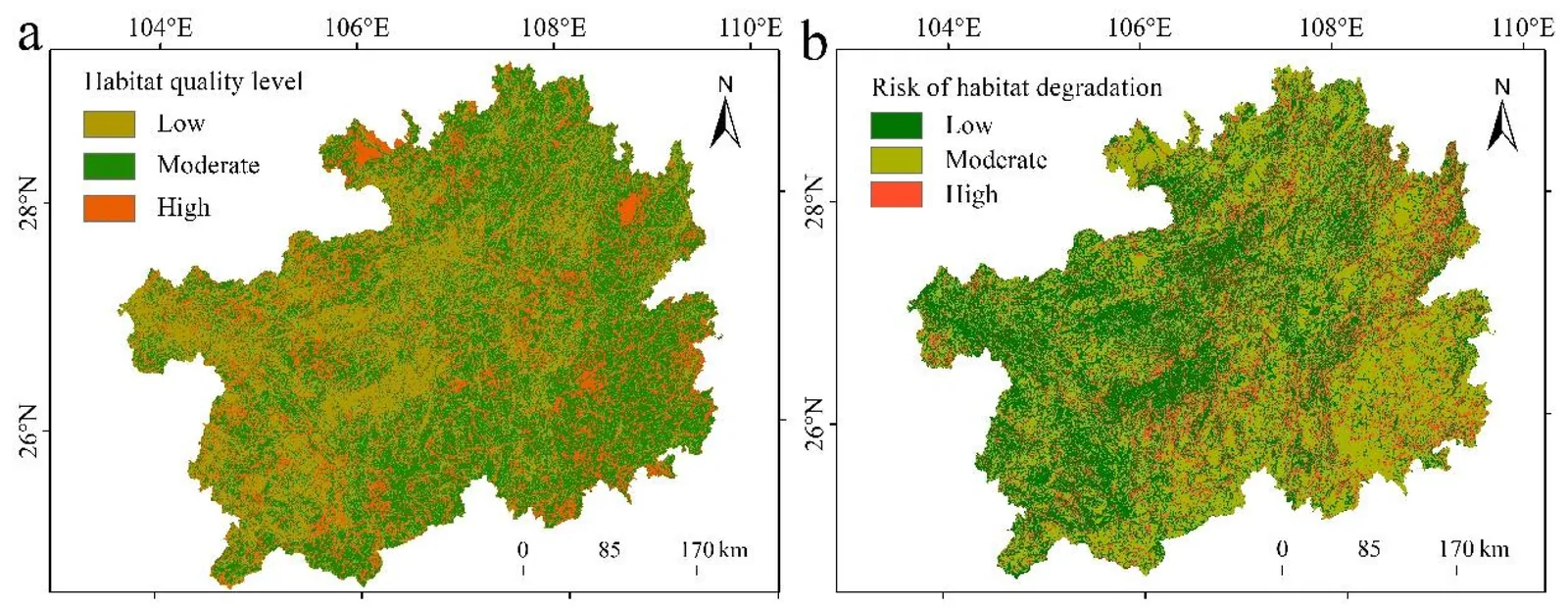
InVEST-derived relative habitat quality and degradation risk across Guizhou Province, southwestern China. (**a**) Spatial distribution of relative habitat quality classified into low, moderate and high levels. (**b**) Spatial distribution of relative habitat degradation risk classified into low, moderate and high levels. Classes were derived from InVEST outputs using the natural breaks method and represent relative land-cover-based habitat condition rather than absolute ecological thresholds.

The InVEST assessment revealed strong spatial heterogeneity in land cover-based relative habitat quality across Guizhou (Figure 3a; Table S5). Moderate-quality areas formed the largest class, covering 81,309 km^2^ (46.2% of the province), followed by low-quality (62,121 km^2^, 35.3%) and high-quality areas (32,668 km^2^, 18.6%). High-quality areas were relatively restricted and discontinuous, occurring mainly in the northern, northeastern, and southeastern mountainous regions, with additional scattered patches in southwestern Guizhou. Low-quality areas were more evident in central-western Guizhou and in locally developed or open landscapes than in other areas.

The relative degradation risk showed a more diffuse spatial pattern than relative habitat quality (Figure 3b; Table S5). Moderate degradation risk covered 76,863 km^2^ (43.7%), followed by low-risk areas (73,019 km^2^, 41.5%) and high-risk areas (26,217 km^2^, 14.9%). Low-risk areas were mainly distributed in western and southwestern Guizhou and in some relatively continuous mountainous areas, whereas high-risk areas occurred as scattered patches and linear clusters, particularly in landscapes associated with built-up land, roads, and agriculture.

### 3.4. Suitability–Quality Mismatch and Protected-Area Representation Gaps

**Figure 4:**
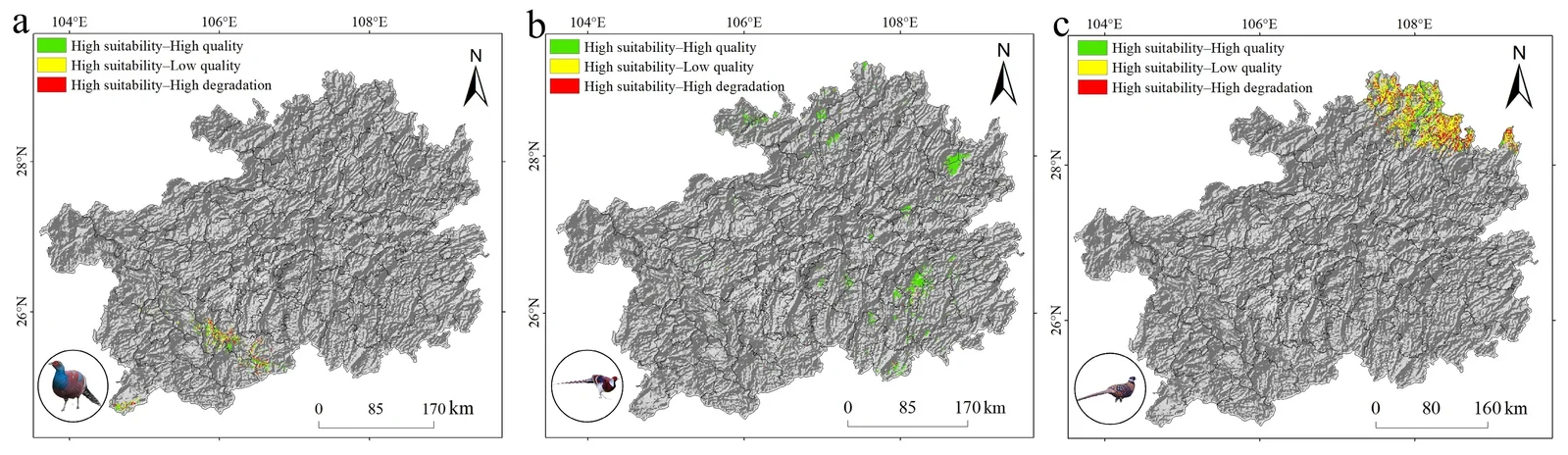
Overlap between predicted high-suitability areas, relative habitat quality and relative degradation risk for three *Syrmaticus* species in Guizhou Province. Spatial overlap is shown for (**a**) *S. humiae*, (**b**) *S. ellioti* and (**c**) *S. reevesii*. Green indicates predicted high-suitability areas overlapping with areas of high relative habitat quality, yellow indicates predicted high-suitability areas overlapping with areas of low relative habitat quality, and red indicates predicted high-suitability areas overlapping with areas of high relative degradation risk.

**Figure 5:**
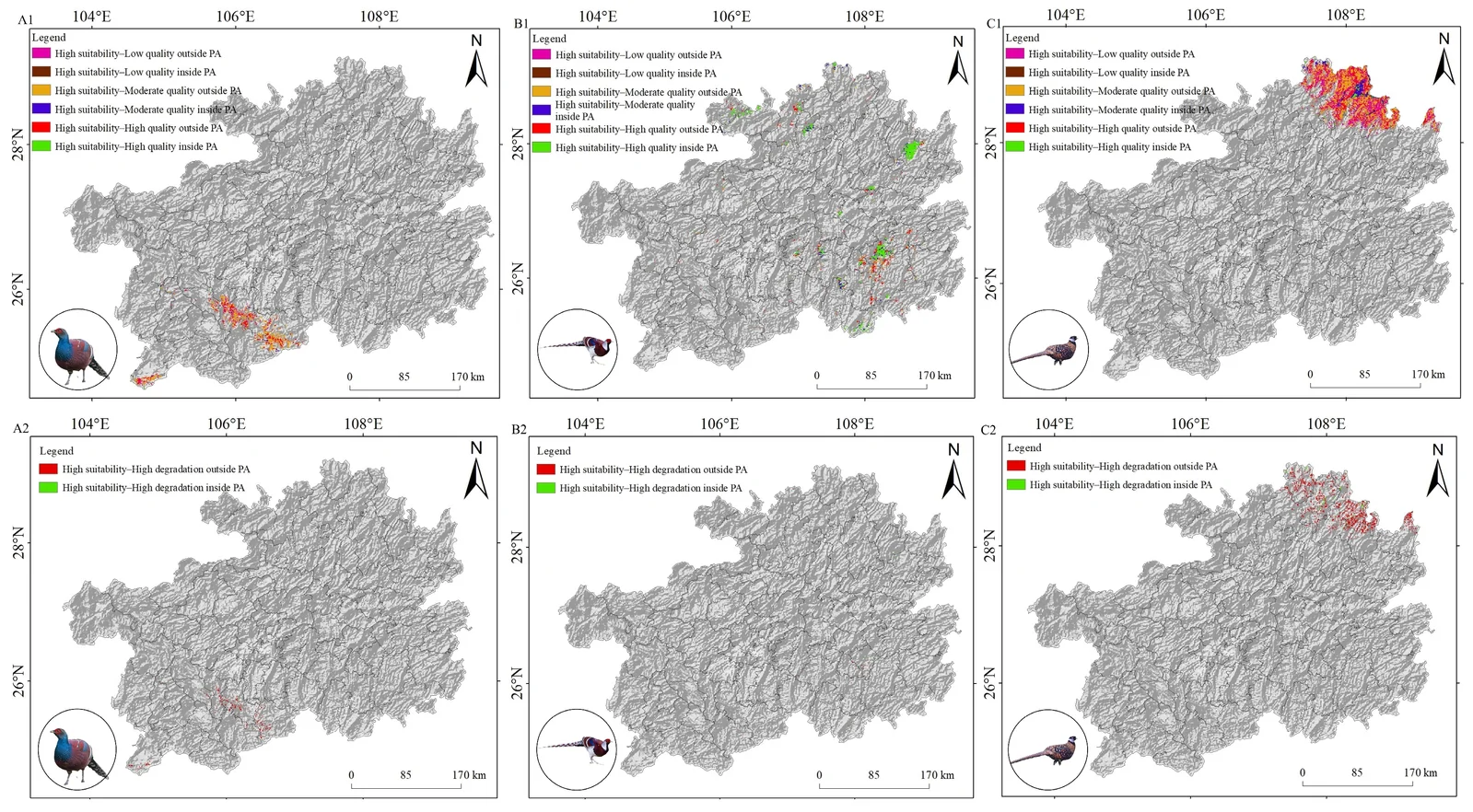
Protected-area representation of predicted high-suitability areas across relative habitat-quality and degradation-risk classes for three *Syrmaticus* species in Guizhou Province. Panels (**A1**–**C1**) show the overlap among predicted high-suitability areas, relative habitat-quality classes and protected areas for *S. humiae* (**A1**), *S. ellioti* (**B1**) and *S. reevesii* (**C1**). Panels (**A2**–**C2**) show the overlap between predicted high-suitability areas with high relative degradation risk and protected areas for the same species. PA indicates protected areas.

Overlay analyses revealed clear species-specific mismatches between predicted high-suitability areas and relative land-cover-based habitat condition (Figure 4; Tables S6 and S7). For *S. humiae*, predicted high-suitability areas overlapped with 573 km^2^ of high relative habitat quality, 474 km^2^ of low relative habitat quality and 295 km^2^ of high relative degradation risk. Protected-area representation of these categories was consistently low: only 24 km^2^ (4.2%) of high-suitability × high-quality areas and 13 km^2^ (4.4%) of high-suitability × high-degradation areas occurred inside protected areas (Figure 5; Tables S6 and S7).

For *S. ellioti*, predicted high-suitability areas showed the strongest correspondence with high relative habitat quality among the three species. These areas overlapped with 2401 km^2^ of high relative habitat quality and only 81 km^2^ of low relative habitat quality. However, 1126 km^2^ also overlapped with high relative degradation risk, indicating that a substantial portion of the predicted high-suitability area was still associated with elevated degradation risk. Protected-area representation was highest for high-suitability × high-quality areas in this species, with 1677 km^2^ (69.9%) inside protected areas and 724 km^2^ (30.2%) outside. In contrast, only 69 km^2^ (6.1%) of its high-suitability × high-degradation areas occurred inside protected areas, leaving 1057 km^2^ (93.9%) outside the current protected-area network (Figure 5; Tables S6 and S7).

*S. reevesii* showed the clearest suitability–quality mismatch. Its predicted high-suitability areas overlapped with 1466 km^2^ of high relative habitat quality, but much larger areas overlapped with low relative habitat quality (3540 km^2^) and high relative degradation risk (1739 km^2^). Protected-area representation was also limited: only 167 km^2^ (11.4%) of high-suitability × high-quality areas and 96 km^2^ (5.5%) of high-suitability × high-degradation areas occurred inside protected areas. Most of these areas therefore remained outside the current protected-area network (Tables S6 and S7). Overall, *S. humiae* and *S. reevesii* showed substantial representation gaps in predicted high-suitability areas, whereas *S. ellioti* showed relatively strong protected-area representation of high-suitability × high-quality areas.

The scenario-based InVEST sensitivity analysis showed that the principal pattern of protected-area representation for high-suitability × high-quality areas was generally retained under alternative parameterizations (Table S9). *S. ellioti* consistently showed the highest protected-area representation of these areas across the Baseline, Higher-condition and Lower-condition scenarios. The absolute extent of high-quality and high-degradation overlaps varied more strongly among parameterizations, particularly under the Lower-condition scenario, in which high-suitability × high-quality areas became very limited for *S. humiae* and *S. reevesii*. These results indicate that the main species-specific representation pattern was relatively robust, although the estimated extent of individual habitat-condition categories remained sensitive to InVEST parameter assumptions.

### 3.5. Protected-Area Representation of Projected Expansion Areas Under Future Climate Scenarios

**Figure 6:**
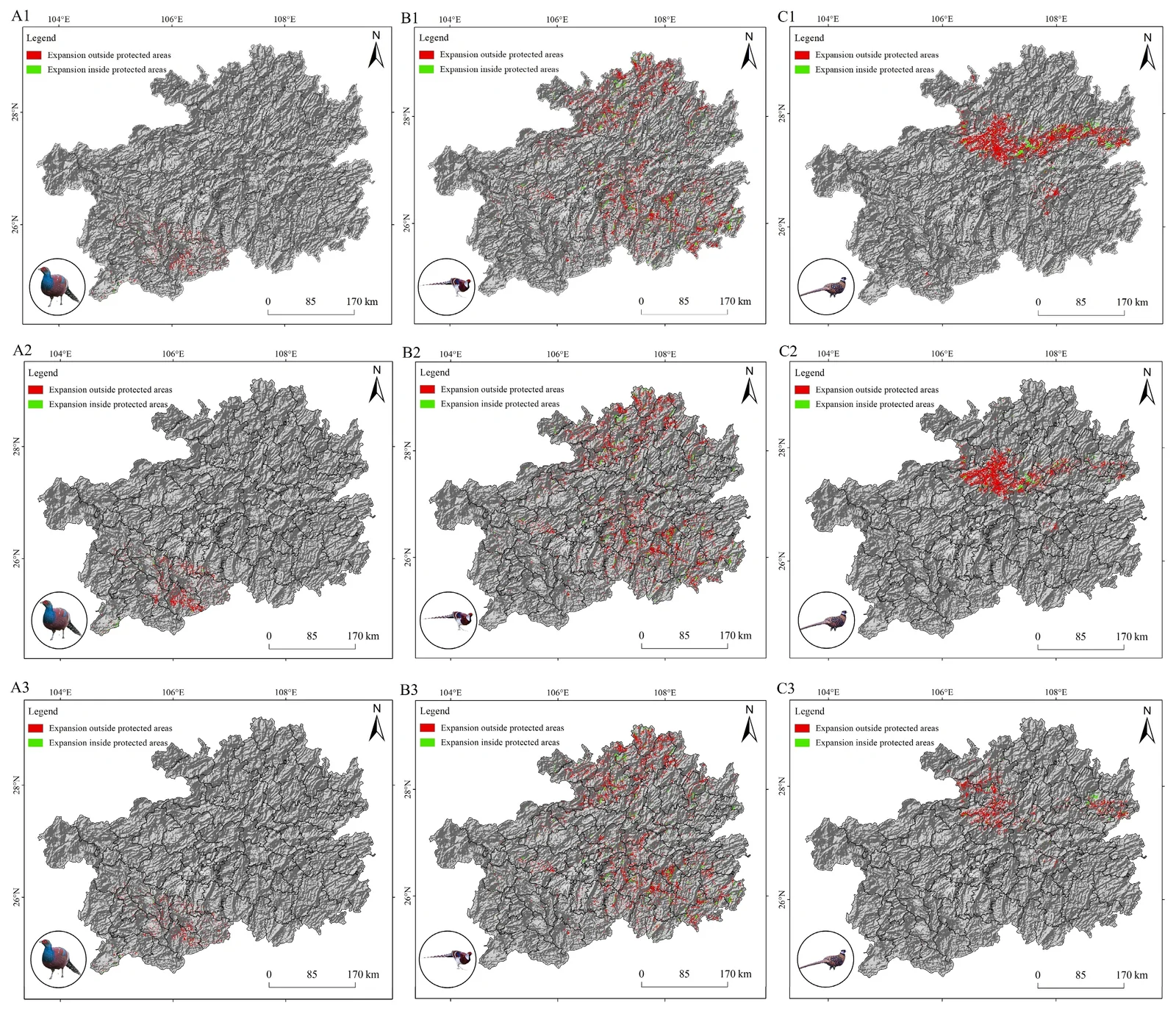
Protected-area representation of projected expansion areas in potential climatic suitability for three *Syrmaticus* species under mid-century climate scenarios in Guizhou Province. Rows represent climate scenarios: SSP126 (first row), SSP245 (second row) and SSP585 (third row). Columns represent species: *S. humiae* (**A1**–**A3**), *S. ellioti* (**B1**–**B3**) and *S. reevesii* (**C1**–**C3**). Expansion areas were identified from binary suitability-change maps by comparing the current suitability projection with the corresponding 2041–2060 projection under each scenario. Red indicates projected expansion areas outside the current protected-area network, whereas green indicates projected expansion areas within protected areas. These expansion areas represent projected gains in potential climatic suitability rather than confirmed future habitat expansion or occupancy.

Across SSP126, SSP245, and SSP585, most projected expansion areas in potential suitability occurred outside the current protected-area network for all three *Syrmaticus* species (Figure 6; Table S8). For *S. humiae*, projected expansion areas ranged from 1051 to 1528 km^2^, of which only 6.6–7.1% were located inside protected areas. Specifically, protected-area coverage was 6.72% under SSP126, 6.61% under SSP245, and 7.14% under SSP585. *S. ellioti* showed the largest projected expansion areas across the three scenarios (10,083–10,119 km^2^), but protected-area representation remained consistently low, ranging from 13.40% to 13.74%. For *S. reevesii*, projected expansion declined from 7828 km^2^ under SSP126 to 5331 km^2^ under SSP245 and 3084 km^2^ under SSP585, while protected-area coverage also decreased from 10.22% to 8.16% and 8.07%, respectively. Overall, the majority of projected expansion areas remained outside the current protected-area network under all three climate scenarios, indicating persistent potential future representation gaps across species.

## 4. Discussion

### 4.1. Species-Specific Habitat Drivers and Model Uncertainty

The contrasting predictor profiles of the three *Syrmaticus* species indicate that habitat suitability in Guizhou is associated with species-specific environmental gradients rather than with a single shared driver. The predicted suitability for *S. humiae* was mainly associated with temperature seasonality, annual mean temperature, elevation, and precipitation-related variables, suggesting a strong climatic and elevational component. This pattern is consistent with evidence that temperature, moisture, and topography jointly influence the distribution of montane birds through their effects on vegetation structure, resource availability, and local environmental conditions [10,49]. The model for *S. reevesii* was also dominated by climatic predictors, especially temperature seasonality and precipitation variables, indicating that its predicted suitability was primarily related to broad climatic gradients.

In contrast, *S. ellioti* was more strongly associated with landscape-condition variables. Soil organic carbon, karst rocky desertification, and the Human Footprint Index contributed most to the model, suggesting closer associations with local habitat conditions, karst-related environmental heterogeneity, and anthropogenic pressure. The contribution of karst rocky desertification to the models of *S. humiae* and *S. ellioti* also suggests that karst-related land degradation may be associated with variation in pheasant habitat suitability in Guizhou. This variable should be interpreted as a broad indicator of exposed bedrock, shallow soils, vegetation degradation, and ecological vulnerability, rather than as a direct measure of fine-scale vegetation structure or realized habitat quality [32,50]. This distinction is important in karst landscapes, where forest fragmentation, shallow soils, agricultural mosaics, and human access can produce sharp local variations in habitat conditions. In such modified landscapes, cumulative human pressure may alter biodiversity patterns even when broad environmental conditions appear suitable [19,20].

Uneven occurrence data among species should be considered when interpreting these patterns. Although MaxEnt is useful for presence-only modelling of rare or incompletely surveyed species, predictions can be sensitive to sample size, background selection, thresholding, and transferability, especially when occurrence records are sparse [11,12,13]. This concern is most relevant for *S. reevesii*, for which only nine spatially filtered occurrence records were available. The leave-one-out analysis indicated moderate sensitivity of the predicted extent to individual occurrence records, with predicted high-suitability areas ranging from 6571 to 14,685 km^2^ (mean = 11,431 ± 3061 km^2^; CV = 26.8%; Table S10), and spatial uncertainty varied across the predicted range (Figure S2). The small sample size may therefore limit the model’s ability to capture the full environmental niche of this species and increase uncertainty in variable importance and the extent and boundaries of predicted suitable areas. Consequently, predicted high-suitability areas for *S. reevesii* should be treated as candidate areas for targeted field verification rather than confirmed core habitats. More broadly, the suitability maps presented here should be used as screening layers for subsequent surveys and integrated habitat assessments rather than as direct evidence of occupied habitats [51].

### 4.2. Potential Suitability Shifts and Climate-Related Representation Gaps

Projected suitable areas increased for all three pheasant species under the 2041–2060 climate scenarios, but these gains represented shifts in potential climatic suitability rather than realized range expansion. Species distribution models estimate environmental similarity to known occurrence conditions, whereas colonization and persistence also depend on dispersal capacity, source populations, habitat continuity, biotic interactions, and human disturbance [14,52]. The projected increases observed here differ from the common expectation that many montane species will undergo upslope shifts or habitat compression under warming [4,53,54]. In Guizhou, these gains indicate that some areas may become climatically similar to current occurrence environments when combined with relatively stable topographic, soil, and karst rocky desertification backgrounds. Their ecological relevance will nevertheless depend on landscape connectivity and local habitat conditions, particularly in karst landscapes where fragmented forests, steep terrain, farmland mosaics, and road networks may constrain movement among suitable patches [36].

The interpretation of future projections also depends on the predictor set used for model transfer. We replaced only the bioclimatic predictors with 2041–2060 climate layers, while retaining relatively stable non-climatic predictors, including topography, soil properties, and karst rocky desertification. Dynamic predictors likely to change substantially in the future, including vegetation productivity, land use/land cover, the Human Footprint Index, and road-related variables, were excluded from future-transfer models because reliable scenario-specific projections were not consistently available. This strategy reduces uncertainty from poorly constrained future dynamic layers but may overestimate functional habitat availability if future land conversion, infrastructure expansion, or increasing human pressure reduces habitat conditions in areas projected to become climatically suitable [17,19]. Across SSP126, SSP245, and SSP585, most projected expansion areas occurred outside the current protected-area network for all three species. Protected-area coverage remained consistently low, ranging from 6.61 to 7.14% for *S. humiae*, 13.40–13.74% for *S. ellioti*, and 8.07–10.22% for *S. reevesii* (Figure 6; Table S8). These results indicate that projected gains in potential suitability are poorly represented within the existing protected-area network and identify areas outside current protection that warrant consideration in climate-adaptation monitoring and adaptive conservation planning [17,31].

### 4.3. Suitability–Quality Mismatch and Habitat-Condition Constraints

High predicted suitability did not always coincide with favourable land-cover-based habitat conditions, underscoring the need to distinguish potential distribution from relative habitat quality in conservation planning. MaxEnt identifies areas with environmental conditions similar to those associated with species occurrences, whereas the InVEST Habitat Quality model estimates relative habitat conditions from land cover, threat sources, and habitat sensitivity [21,43]. Combining these outputs provides a more conservative basis for identifying conservation priorities than relying on suitability maps alone, because areas predicted as suitable may still be degraded, fragmented, or exposed to human disturbance. This agrees with conservation planning studies showing that priority areas can change substantially when land-use change, habitat loss, or model uncertainty are considered alongside species distribution predictions [16,51]. Recent MaxEnt–InVEST applications have also shown that suitable areas and high-quality habitats may be only partly congruent, and that their overlap can provide more practical information for identifying conservation priorities under environmental change [55].

The three pheasant species showed contrasting relationships between predicted suitability and relative habitat condition. *S. ellioti* showed the strongest apparent correspondence between predicted high suitability and high relative habitat quality, with 2401 km^2^ of high-suitability × high-quality overlap but only 81 km^2^ overlapping low relative habitat quality. In contrast, *S. reevesii* showed the clearest suitability–quality mismatch: its predicted high-suitability areas overlapped with 1466 km^2^ of high relative habitat quality, whereas substantially larger areas overlapped with low relative habitat quality (3540 km^2^) and high relative degradation risk (1739 km^2^). This pattern should nevertheless be interpreted cautiously because the underlying suitability model for *S. reevesii* was based on only nine occurrence records and showed moderate sensitivity to individual records. For *S. humiae*, the area simultaneously classified as highly suitable and of high relative habitat quality was limited in absolute extent, indicating that relatively few areas combined both favourable suitability and habitat-condition attributes.

These mismatches have direct implications for prioritization. Predicted high-suitability × high-quality areas provide the strongest candidates for habitat prioritization, whereas predicted high-suitability areas overlapping low relative habitat quality require additional assessment to determine whether they reflect actual habitat limitation, recent degradation, or potentially restorable habitat. Areas combining high predicted suitability with high relative degradation risk may instead require restoration, threat mitigation, or disturbance management [56]. Thus, for forest-associated pheasants in karst landscapes, SDM-derived suitability is best used as an initial screening layer, with conservation value evaluated jointly with relative habitat quality, degradation risk, and protected-area representation [2].

### 4.4. Protected-Area Representation Gaps and Conservation Priorities

Protected-area representation differed strongly among the three pheasant species once predicted suitability was combined with relative habitat quality and degradation risk. The current protected-area network showed the highest representation for *S. ellioti*, with 69.9% of its predicted high-suitability × high-quality areas occurring within protected areas. In contrast, *S. humiae* and *S. reevesii* showed substantial representation gaps, with only 4.2% and 11.4% of their predicted high-suitability × high-quality areas, respectively, occurring within protected areas. These results show that protected-area representation cannot be inferred from total protected extent alone, but depends on whether protected areas spatially represent the habitat-condition categories most relevant to target species [22,26,57]. Importantly, such spatial representation should not be interpreted as evidence of effective management or protection.

Overlaying high-suitability areas with relative degradation risk further distinguished representation gaps from restoration or management gaps. Under the baseline parameterization, most predicted high-suitability × high-degradation areas occurred outside protected areas, with protected-area coverage of 4.4% for *S. humiae*, 6.1% for *S. ellioti*, and 5.5% for *S. reevesii*. The scenario-based InVEST sensitivity analysis showed that the main species-specific pattern of high-suitability × high-quality representation was generally retained under alternative parameterizations, with *S. ellioti* consistently showing the highest protected-area representation (Table S9). However, the absolute extent of high-quality and high-degradation overlaps varied among parameterizations, indicating sensitivity of individual habitat-condition estimates to user-defined InVEST assumptions. These results support distinguishing current representation gaps from areas where restoration or management interventions may be more appropriate [28,56].

Together with the future protected-area analysis, these findings indicate three complementary conservation gaps for Guizhou’s rare pheasants: current representation gaps in unprotected high-suitability × high-quality areas, restoration or management gaps in high-suitability × high-degradation areas, and potential future representation gaps where projected expansion occurs outside the existing protected-area network.

### 4.5. Limitations and Conservation Implications

This study has several limitations. Occurrence records were uneven among species, particularly for *S. reevesii*. Although the province-wide camera-trap network and 1 km spatial thinning reduced spatial clustering and sampling bias, residual bias associated with uneven camera deployment cannot be completely excluded. The leave-one-out analysis further indicated moderate sensitivity of the predicted extent for *S. reevesii* to individual occurrence records; therefore, its predicted suitability and protected-area representation should be treated as spatial hypotheses requiring further verification. Future projections represented potential climatic suitability under static non-climatic constraints because future land use, vegetation productivity, infrastructure expansion, restoration, and human pressure were not explicitly modelled. In addition, InVEST outputs should be interpreted as relative land-cover-based habitat conditions rather than absolute or species-specific realized habitat quality. Although the scenario-based sensitivity analysis indicated that the principal species-specific representation pattern was generally retained, the absolute extent of habitat-condition categories varied among alternative parameterizations, and field validation would further strengthen their ecological interpretation [21,43]. Finally, the binary protected-area overlay represents spatial or nominal coverage and does not account for differences among protected-area categories in legal status, management intensity, enforcement, or conservation effectiveness.

Despite these uncertainties, the integrated MaxEnt–InVEST–protected-area framework provides a practical screening tool for adaptive conservation planning [2]. Unprotected areas where predicted high suitability coincides with high relative habitat quality warrant priority consideration for field surveys and conservation assessment, whereas predicted high-suitability areas associated with low relative habitat quality or high relative degradation risk may require restoration, disturbance control, or habitat improvement before being considered conservation cores. Conservation beyond existing protected-area networks may therefore be necessary to improve species and habitat representation [57]. By distinguishing candidate areas primarily associated with protection, restoration, and monitoring needs, this framework can help provincial conservation agencies refine spatial priorities for rare pheasants in karst mountain landscapes under ongoing environmental change.

## 5. Conclusions

This study demonstrates that predicted habitat suitability alone is insufficient for identifying conservation priorities for rare pheasants in the karst landscapes of Guizhou Province. The three *Syrmaticus* species differed in their environmental associations, projected changes in potential suitability, habitat-condition patterns, and representation within the existing protected-area network. Under the 2041–2060 climate scenarios, projected potential suitable areas increased for all three species, although these changes represent potential climatic suitability rather than confirmed range expansion, and most projected expansion areas under SSP126, SSP245, and SSP585 occurred outside protected areas. Integrating MaxEnt, InVEST, and protected-area overlays further showed that predicted high suitability did not always coincide with favourable land-cover-based habitat condition. *S. reevesii* showed the clearest suitability–quality mismatch, whereas *S. ellioti* had the highest protected-area representation of high-suitability × high-quality areas. The principal species-specific representation pattern was generally retained under alternative InVEST parameterizations, although the absolute extent of habitat-condition categories remained parameter-sensitive. Unprotected high-suitability × high-quality areas should therefore be prioritized for field verification and conservation assessment, while high-suitability areas associated with low relative habitat quality or high degradation risk require restoration and threat-mitigation evaluation. Overall, this integrated framework provides a practical basis for distinguishing candidate areas for protection, restoration, and climate-adaptation monitoring in heterogeneous karst landscapes.

## Figures and Tables

**Table 1:** Relative contributions of environmental variables to MaxEnt models for three *Syrmaticus* species.

Full Variable	Variable Code	*S. humiae*	*S. ellioti*	*S. reevesii*
Temperature seasonality	bio4	47.2	5.3	51.1
Annual mean temperature	bio1	16.1	-	-
Precipitation of driest month	bio14	7.2	1.6	20.1
Annual precipitation	bio12	3.5	-	19.6
Elevation	elevation	10.8	-	-
Slope	slope	-	2.3	-
Terrain roughness	terrain	-	7.1	-
Rocky desertification	rocky	11	18.5	-
Cumulative gross primary productivity	gpp_cum	-	2.8	-
Land use	lucc	2.4	-	-
Distance to roads	disroad	-	5.6	-
Human Footprint Index	hfp	-	13.3	0.4
Soil organic carbon	soil_oc	-	43.4	-
Soil pH	soil_ph	1.8	-	8.7
Soil clay content	soil_clay	-	-	0.1

Note: Values indicate the percentage contribution (%) of each environmental variable to the MaxEnt model. “-” indicates that the variable was not retained in the final model for the species.

## Data Availability

The data supporting the findings of this study are available from the corresponding author upon reasonable request. Exact camera-trap coordinates and species occurrence locations are not publicly available because the focal species are threatened, and disclosure of precise locations could increase the risks of wildlife disturbance or illegal hunting. Environmental predictor datasets are available from the original sources listed in Table S1.

## Supplementary Materials

The following supporting information can be downloaded at: https://www.mdpi.com/article/10.3390/ani16172753/s1, Figure S1: Location of Guizhou Province in southwestern China and distribution of camera-trap survey sites (n = 1573) and spatially filtered occurrence records of *Syrmaticus humiae* (n = 65), *S. ellioti* (n = 27), and *S. reevesii* (n = 9) retained after 1 km spatial thinning for MaxEnt modelling; Figure S2: Spatial uncertainty of MaxEnt predictions for *Syrmaticus reevesii* under leave-one-out sensitivity analysis. The map shows the spatial standard deviation of cloglog suitability predictions across nine leave-one-out MaxEnt models. In each model, one of the nine spatially filtered occurrence records was excluded and the remaining eight records were used for model fitting, while all environmental predictors and model settings were kept unchanged. Higher standard deviation values indicate greater sensitivity of predicted suitability to the removal of individual occurrence records, whereas lower values indicate greater consistency among leave-one-out predictions. Purple circles indicate the nine spatially filtered occurrence sites of *S. reevesii* used in the sensitivity analysis; Table S1: Environmental predictors used in MaxEnt modelling [58,59,60,61]; Table S2: Habitat suitability scores and sensitivity values of land-cover classes used in the InVEST Habitat Quality model; Table S3: Threat factors and parameter settings used in the InVEST Habitat Quality model; Table S4: Area transitions of projected suitable areas under 2041–2060 climate scenarios; Table S5: Area and proportion of relative habitat quality and degradation-risk classes in Guizhou Province; Table S6: Protected-area coverage of predicted high-suitability areas across relative habitat-quality classes; Table S7: Protected-area coverage of predicted high-suitability areas with high relative degradation risk; Table S8: Protected-area coverage of projected expansion areas in potential suitability under SSP126, SSP245, and SSP585; Table S9: Sensitivity of protected-area representation of high-suitability areas to alternative InVEST parameterizations; Table S10: Leave-one-out sensitivity of predicted high-suitability area for *Syrmaticus reevesii*.
