# Supplementary material for: Beyond Suitability: Mapping Current and Projected Conservation Gaps for Three Rare Pheasants in Karst Landscapes of Southwest China
Source: Animals (Basel). 2026 Sep 2;16(17):2753. doi: 10.3390/ani16172753 (PMC13564796; doi:10.3390/ani16172753)
Supplement: Supplementary file 1 [file animals-16-02753-s001.zip › animals-4500359-supplementary.pdf]

Supplementary figures and tables

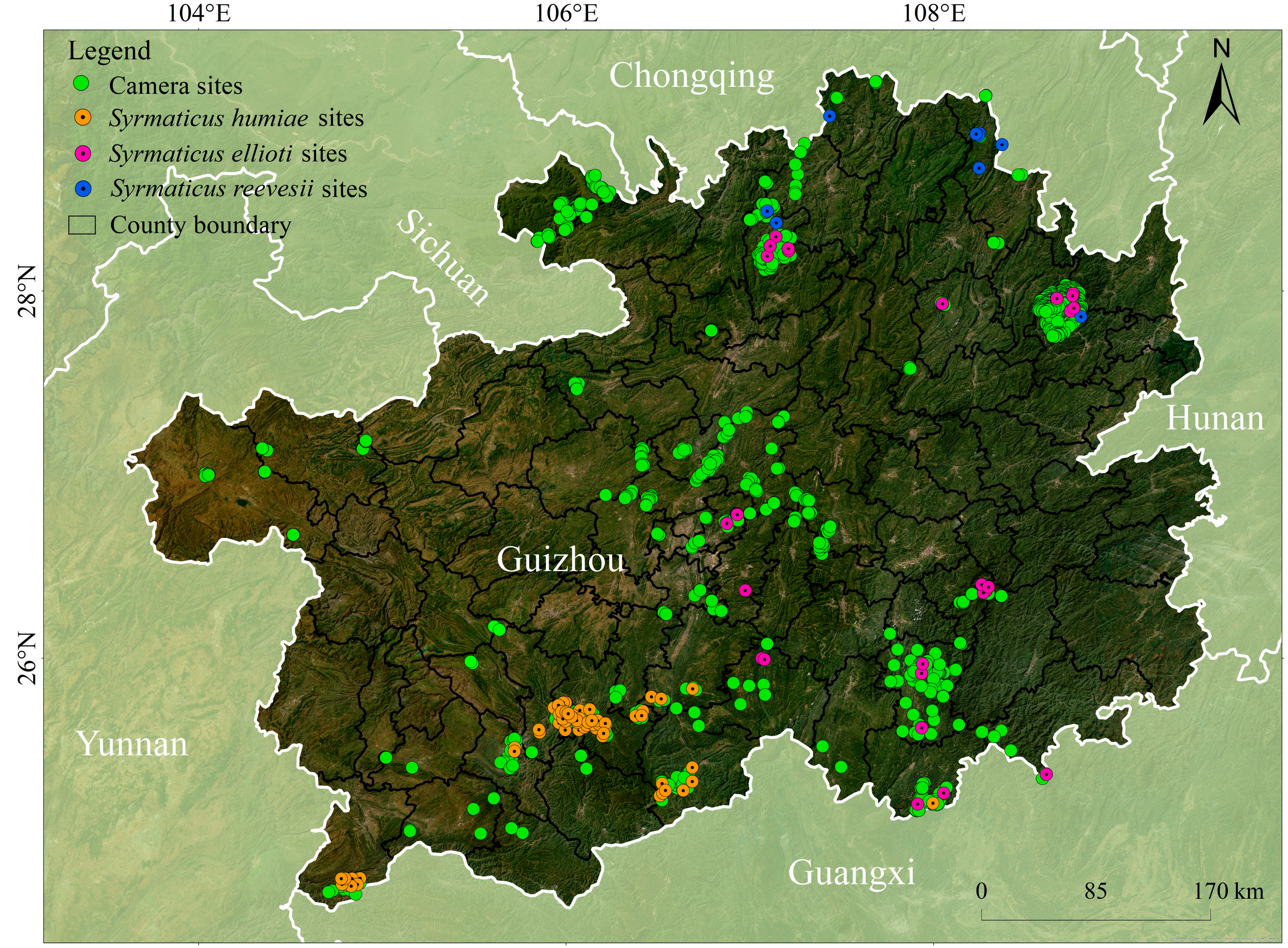

Fig. S1 Location of Guizhou Province in southwestern China and distribution of camera-trap survey sites (n = 1,573) and spatially filtered occurrence records of *Syrmaticus humiae* (n = 65), *S. ellioti* (n = 27) and *S. reevesii* (n = 9) retained after 1km spatial thinning for MaxEnt modelling.

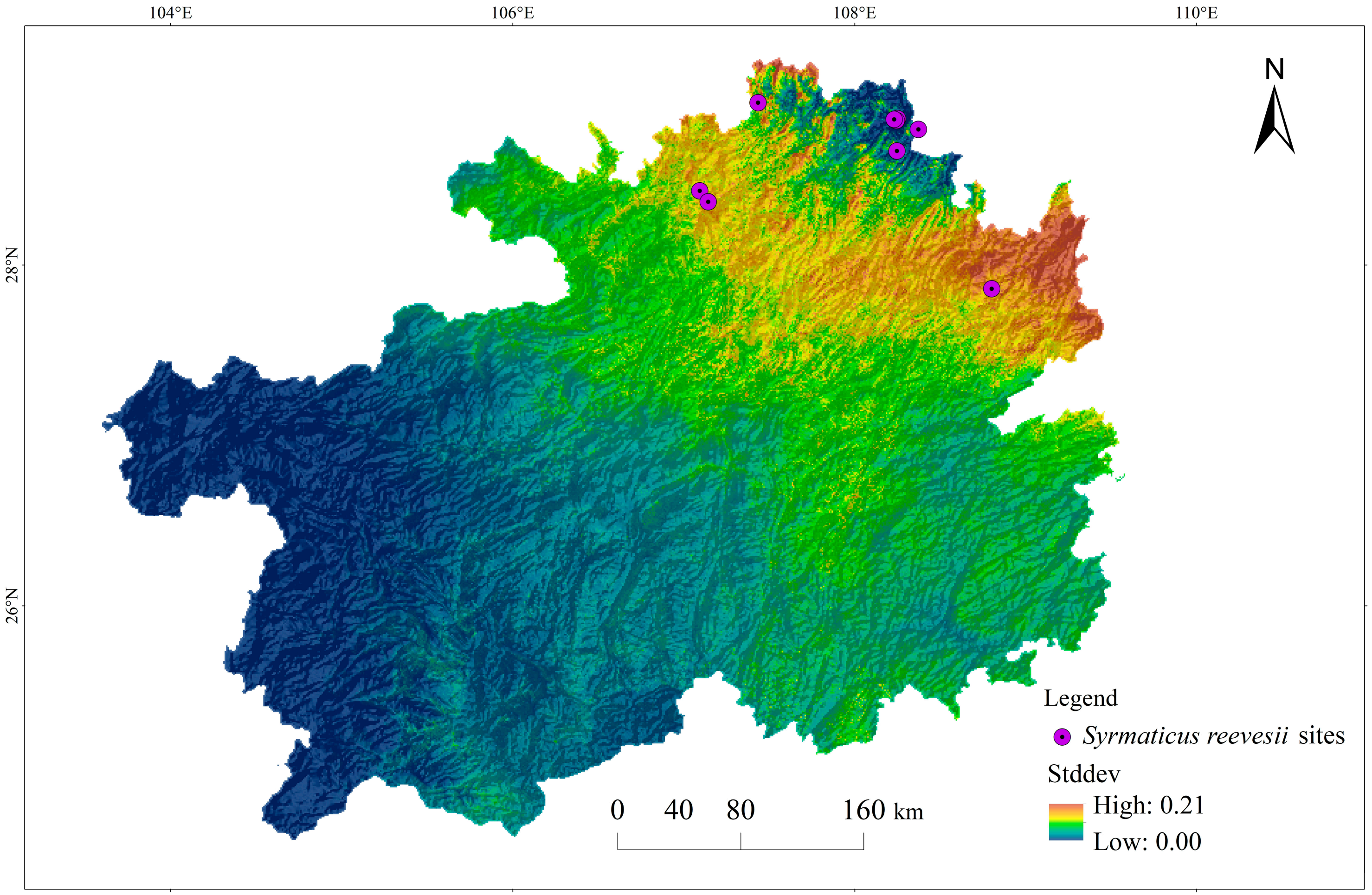

Fig. S2 Spatial uncertainty of MaxEnt predictions for *Syrmaticus reevesii* under leave-one-out sensitivity analysis. The map shows the spatial standard deviation of cloglog suitability predictions across nine leave-one-out MaxEnt models. In each model, one of the nine spatially filtered occurrence records was excluded and the remaining eight records were used for model fitting, while all environmental predictors and model settings were kept unchanged. Higher standard deviation values indicate greater sensitivity of predicted suitability to the removal of individual occurrence records, whereas lower values indicate greater consistency among leave-one-out predictions. Purple circles indicate the nine spatially filtered occurrence sites of *S. reevesii* used in the sensitivity analysis.

Table S1. Environmental predictors used in MaxEnt modelling.

Category	Variable	Code	Source	Native resolution	Future projection
Climate	Annual mean temperature	bio1	WorldClim v2.1	30 arc-sec	Yes
Climate	Temperature seasonality	bio4	WorldClim v2.1	30 arc-sec	Yes
Climate	Annual precipitation	bio12	WorldClim v2.1	30 arc-sec	Yes
Climate	Precipitation of driest month	bio14	WorldClim v2.1	30 arc-sec	Yes
Topography	Elevation	elevation	WorldClim DEM	30 arc-sec	Yes
Topography	Slope	slope	Derived from DEM	30 arc-sec	Yes
Topography	Terrain roughness	terrain	Copernicus DEM	30 m	Yes
Productivity	Cumulative gross primary productivity	gpp_cum	Dynamic Habitat Index (DHI) dataset for China [58]	500 m	No
Land cover	Land-use/land-cover type	lucc	China Land Cover Dataset (CLCD) [59]	30 m	No
Human disturbance	Human Footprint Index	hfp	Annual Human Footprint dataset [60]	1 km	No
Human disturbance	Distance to roads	disroad	OpenStreetMap / Geofabrik	Vector	No
Soil	Soil organic carbon	soil_oc	CSDLv2 [61]	1 km	Yes
Soil	Soil pH	soil_ph	CSDLv2	1 km	Yes
Soil	Soil clay content	soil_clay	CSDLv2	1 km	Yes
Karst environment	Rocky desertification intensity	rocky	Karst Rocky Desertification Dataset of Southwest China [50]	Original vector/raster	Yes

Note: Predictors were selected to represent ecological gradients relevant to forest-associated pheasants. Climate variables describe thermal conditions, seasonal variability, moisture availability and drought stress; topographic variables represent elevational gradients, slope-related accessibility and terrain heterogeneity; cumulative gross primary productivity serves as a proxy for vegetation productivity and potential food availability; land-use/land-cover represents habitat composition and vegetation structure; Human Footprint Index and distance to roads describe cumulative anthropogenic pressure, road-related disturbance, accessibility and hunting risk; soil variables represent fertility, chemical conditions and water-holding capacity that indirectly affect plant communities; and karst rocky desertification indicates karst-related land degradation and ecological vulnerability. Future projection indicates whether a predictor was retained in future-transfer models.

Table S2. Habitat suitability scores and sensitivity values of land-cover classes used in the InVEST Habitat Quality model.

Land-cover class	Habitat suitability	Sensitivity to built-up land	Sensitivity to roads	Sensitivity to cropland	Sensitivity to bare/degraded land
Cropland	0.20	0.15	0.25	0.10	0.15
Forest	1.00	0.95	0.85	0.65	0.85
Shrubland	0.85	0.85	0.75	0.60	0.80
Grassland	0.45	0.55	0.60	0.45	0.65
Water	0.05	0.10	0.10	0.05	0.10
Bare land	0.00	0.00	0.00	0.00	0.00
Built-up land	0.00	0.00	0.00	0.00	0.00

Note: Habitat suitability and sensitivity values range from 0 to 1. Higher habitat suitability values indicate a greater potential habitat value for forest-associated pheasants. Higher sensitivity values indicate stronger expected negative responses of a land-cover class to a given threat factor, and vice versa. Bare land and built-up land were treated as non-habitat classes and therefore assigned zero habitat suitability and zero sensitivity.

Table S3. Threat factors and parameter settings used in the InVEST Habitat Quality model.

Threat factor	Ecological interpretation	Maximum threat distance (m)	Weight	Decay function
Built-up land	Construction pressure, settlement disturbance and increased human activity	1,500	1.00	Exponential
Roads	Road-related disturbance, increased accessibility and potential mortality risk	1,000	0.85	Exponential
Cropland	Agricultural conversion and lower-intensity matrix effects around forest patches	1,000	0.50	Linear
Bare/degraded land	Exposed surfaces, vegetation degradation and reduced local habitat condition	1,000	0.60	Exponential

Note: The maximum threat distance indicates the spatial range over which each threat factor was assumed to affect habitat quality. Threat weights ranged from 0 to 1, with higher values indicating stronger negative effects. The decay function describes how the influence of each threat decreases with distance from the threat source.

Table S4. Area transitions of projected suitable areas under 2041-2060 climate scenarios.

Species	Scenario	Stable unsuitable area (km²)	Expansion area (km²)	Contraction area (km²)	Stable suitable area (km²)
S. humiae	SSP126	217,516	1,071	429	9,336
S. humiae	SSP245	217,059	1,528	550	9,215
S. humiae	SSP585	217,536	1,051	541	9,224
S. ellioti	SSP126	194,834	10,119	5,028	15,457
S. ellioti	SSP245	194,870	10,083	5,007	15,478
S. ellioti	SSP585	194,847	10,106	5,074	15,411
S. reevesii	SSP126	181,471	7,828	1,189	38,760
S. reevesii	SSP245	183,968	5,331	1,018	38,931
S. reevesii	SSP585	186,215	3,084	1,144	38,805

Note: Stable unsuitable areas indicate pixels unsuitable in both current and future projections; expansion areas indicate pixels unsuitable at present but suitable under future climate scenarios; contraction areas indicate pixels suitable at present but unsuitable in the future; stable suitable areas indicate pixels suitable in both periods. Future changes represent projected potential climatic suitability rather than confirmed future habitat occupancy.

Table S5. Area and proportion of relative habitat quality and degradation-risk classes in Guizhou Province.

InVEST output	Class	Area (km²)	Proportion (%)
Relative habitat quality	High	32,668	18.6
Relative habitat quality	Moderate	81,309	46.2
Relative habitat quality	Low	62,121	35.3
Relative degradation risk	High	26,217	14.9
Relative degradation risk	Moderate	76,863	43.7
Relative degradation risk	Low	73,019	41.5

Note: Classes were derived from InVEST habitat quality and degradation outputs using the natural breaks method. Values represent relative land-cover-based habitat condition and degradation risk, not absolute ecological thresholds.

Table S6. Protected-area coverage of predicted high-suitability areas across relative habitat-quality classes.

Species	Relative habitat-quality class within predicted high-suitability areas	Outside protected areas (km²)	Inside protected areas (km²)	Total area (km²)	Protected-area coverage (%)
S. humiae	Low	455	19	474	4.01
S. humiae	Moderate	1,340	58	1,398	4.15
S. humiae	High	549	24	573	4.19
S. ellioti	Low	56	25	81	30.86
S. ellioti	Moderate	564	538	1,102	48.82
S. ellioti	High	724	1,677	2,401	69.85
S. reevesii	Low	3,351	189	3,540	5.34
S. reevesii	Moderate	4,376	491	4,867	10.09
S. reevesii	High	1,299	167	1,466	11.39

Note: Values indicate the area of overlap between predicted high-suitability areas from MaxEnt models, relative habitat-quality classes from the InVEST Habitat Quality model and the protected-area network. Protected-area coverage was calculated as the proportion of each habitat-quality class within predicted high-suitability areas that occurred inside protected areas. Relative habitat-quality classes represent land-cover-based habitat condition and should not be interpreted as absolute ecological thresholds.

Table S7. Protected-area coverage of predicted high-suitability areas with high relative degradation risk.

Species	Outside protected areas (km²)	Inside protected areas (km²)	Total area (km²)	Protected-area coverage (%)
S. humiae	282	13	295	4.41
S. ellioti	1,057	69	1,126	6.13
S. reevesii	1,643	96	1,739	5.52

Note: Values indicate the area of overlap between predicted high-suitability areas, areas of high relative degradation risk from the InVEST Habitat Quality model and the protected-area network. Protected-area coverage was calculated as the proportion of predicted high-suitability x high-degradation areas occurring inside protected areas. Relative degradation risk represents land-cover-based degradation pressure and should not be interpreted as an absolute measure of habitat degradation.

Table S8. Protected-area coverage of projected expansion areas in potential suitability under SSP126, SSP245, and SSP585.

Scenario	Species	Expansion outside protected areas (km²)	Expansion inside protected areas (km²)	Total projected expansion area (km²)	Protected-area coverage (%)
SSP126	*S. humiae*	999	72	1,071	6.72
SSP126	*S. ellioti*	8,729	1,390	10,119	13.74
SSP126	*S. reevesii*	7,028	800	7,828	10.22
SSP245	*S. humiae*	1,427	101	1,528	6.61
SSP245	*S. ellioti*	8,730	1,353	10,083	13.42
SSP245	*S. reevesii*	4,896	435	5,331	8.16
SSP585	*S. humiae*	976	75	1,051	7.14
SSP585	*S. ellioti*	8,752	1,354	10,106	13.4
SSP585	*S. reevesii*	2,835	249	3,084	8.07

Note: Projected expansion areas indicate pixels classified as unsuitable under current conditions but suitable under the corresponding 2041–2060 SSP126, SSP245, or SSP585 projection. Protected-area coverage was calculated as the proportion of projected expansion areas located within the current protected-area network. These areas represent projected expansion in potential climatic suitability under each climate scenario rather than confirmed future habitat expansion, range expansion, or species occupancy.

Table S9. Sensitivity of protected-area representation of high-suitability areas to alternative InVEST parameterizations.

Scenario	Species	HS × High HQ outside PA (km²)	HS × High HQ inside PA (km²)	HS × High HQ total (km²)	HQ PA coverage (%)	HS × High HD outside PA (km²)	HS × High HD inside PA (km²)	HS × High HD total (km²)	HD PA coverage (%)
Baseline	*S. humiae*	549	24	573	4.19	282	13	295	4.41
Baseline	*S. ellioti*	724	1,677	2,401	69.85	1,057	69	1,126	6.13
Baseline	*S. reevesii*	1,299	167	1,466	11.39	1,643	96	1,739	5.52
Higher condition	*S. humiae*	1,815	78	1,893	4.12	23	4	27	14.81
Higher condition	*S. ellioti*	1,257	2,206	3,463	63.7	5	2	7	28.57
Higher condition	*S. reevesii*	4,850	595	5,445	10.93	218	10	228	4.39
Lower condition	*S. humiae*	4	1	5	20	1,506	64	1,570	4.08
Lower condition	*S. ellioti*	6	14	20	70	722	760	1,482	51.28
Lower condition	*S. reevesii*	4	0	4	0	4,831	523	5,354	9.77

Note: HS denotes predicted high-suitability areas derived from MaxEnt; HQ denotes high relative habitat quality; HD denotes high relative degradation risk; and PA denotes the current protected-area network. The Baseline scenario represents the original InVEST parameterization used in the main analysis. For the Higher-condition scenario, habitat suitability scores were increased by 20%, whereas threat-sensitivity values, threat weights, and maximum threat distances were decreased by 20%. For the Lower-condition scenario, habitat suitability scores were decreased by 20%, whereas threat-sensitivity values, threat weights, and maximum threat distances were increased by 20%. Parameter values constrained to the interval 0–1 were capped at 1.0 where necessary, while all other model inputs and settings were held constant. PA coverage was calculated as the proportion of each HS × High HQ or HS × High HD overlap located within the protected-area network. Because the HS × High HQ overlap under the Lower-condition scenario was very small for *S. humiae* (5 km²) and *S. reevesii* (4 km²), the corresponding PA coverage percentages should be interpreted together with the absolute overlap areas.

Table S10. Leave-one-out sensitivity of predicted high-suitability area for *Syrmaticus reevesii*.

Statistic / Fold	Fold 1	Fold 2	Fold 3	Fold 4	Fold 5	Fold 6	Fold 7	Fold 8	Fold 9	Mean	SD	Minimum	Maximum	CV (%)
Predicted high-suitability area (km²)	6,571	10,892	14,210	14,271	7,965	12,824	14,685	12,938	8,527	11,431	3,061	6,571	14,685	26.8

Note: Nine leave-one-out MaxEnt models were fitted for *Syrmaticus reevesii*. In each model, one of the nine spatially filtered occurrence records was excluded and the remaining eight records were used for model fitting, while all environmental predictors and model settings were held constant. High-suitability areas were delineated using the same thresholding procedure as in the main analysis. SD represents the sample standard deviation across the nine models, and CV was calculated as SD divided by the mean predicted high-suitability area × 100. These results quantify the sensitivity of the predicted extent to individual occurrence records rather than providing independent estimates of the species' realized distribution.
